# The Role of Physical Examinations in Psychiatry as Illustrated in a Case of Neuroleptic Malignant Syndrome Versus Viral Encephalitis: A Case Report and Literature Review

**DOI:** 10.7759/cureus.4840

**Published:** 2019-06-05

**Authors:** Michael Esang, Sabina Goldstein, Ravina Dhami

**Affiliations:** 1 Psychiatry and Behavioral Sciences, Nassau University Medical Center, East Meadow, USA; 2 Miscellaneous, American University of the Caribbean, Cupecoy, SXM

**Keywords:** psychiatry, neuropsychiatric symptoms, neuroleptic malignant syndrome, herpes simplex encephalitis, physical examination

## Abstract

Although a standard psychiatric evaluation includes a physical examination, there are no guidelines on the components of a comprehensive physical examination during psychiatric patient encounters. The mental status examination is frequently considered the psychiatric physical examination equivalent. We report a 59-year-old male inpatient on a medical unit who had hyperthermia, an altered mental status, muscle rigidity, and elevated white blood cell count and creatine phosphokinase level. He had been taking risperidone 1 mg orally every 12 hours for two months. His primary treatment team suspected Neuroleptic Malignant Syndrome (NMS), but the consulting psychiatrist detected equivocal findings on physical examination and recommended a broader differential diagnosis. Further investigations revealed the possibility of an infection. The patient was positive for immunoglobulin G (IgG) antibodies to HSV-1 and HSV-2 on cerebrospinal fluid analysis. He was then treated for Herpes Simplex Encephalitis (HSE) with an oral course of acyclovir. Although NMS was low in the diagnostic ranking, given the possibility of an atypical form and the lethality of this condition if untreated, he also received intravenous lorazepam at 2 mg every six hours. He experienced full resolution of his symptoms and was stable for discharge. HSE and NMS are two examples of neuropsychiatric disorders with similar presenting symptoms.

HSE frequently presents with predominantly psychiatric symptoms, such as paranoia, hallucinations, and an altered mental status. Consequently, it is typically not the first diagnosis that comes to mind, especially when these symptoms occur in a patient already being treated by a psychiatrist. Confirmation bias is the tendency for an individual to focus on the information that aligns with one’s preconceptions and to ignore information that defies it. Due to this bias, physicians may attribute all symptoms of a known psychiatric patient to a psychiatric cause, instead of considering an organic etiology. In this case, the evaluation by the psychiatrist was crucial in guiding the treatment team to a diagnosis of HSE. This is important since a delayed treatment of HSE can be fatal. The literature review reveals a general consensus among psychiatrists on the value of physical examinations in patient care. In spite of this, the majority of psychiatrists seldom perform physical examinations due to concerns over skill atrophy and the potential that doing so may change the therapeutic dynamic. Others have disputed these claims and have argued that physical examinations in a psychiatric setting will not only strengthen the perception of a psychiatrist as a physician by the patient but will also allow for better care of psychiatrically ill patients. Psychiatrists should remember that they are oftentimes the sole healthcare provider for psychiatric patients and that these patients may not have the access to primary care physicians and may lack the ability to explain their symptoms or advocate for themselves. Therefore, incorporating an emphasis on performing physical examinations during psychiatry residency training and in continuing medical education programs for psychiatrists is essential.

## Introduction

As the branch of medicine that deals with disorders of the mind, psychiatry may be seen as a medical discipline in which its practitioners are not frequently tasked to perform physical examinations. This, however, could not be further from the truth, especially in the context of patients with severe mental illness (SMI). It has been established that the life expectancy of Americans with SMI is about 14 to 32 years shorter than the general population, and there is further evidence that this trend will continue in the absence of any meaningful interventions [1-2]. Rather than diminish the need for physical examinations, patient encounters in psychiatry often require the exclusion (or inclusion) of physical disease as a cause or comorbid condition in the patient’s pathologic state. In fact, in a policy statement from the American College of Emergency Physicians, physical examination, in conjunction with obtaining previous medical and psychiatric history, is recommended to guide patient evaluation [3]. Two conditions that highlight this importance are neuroleptic malignant syndrome (NMS) and viral encephalitis (VE).

NMS is a life-threatening neuropsychiatric emergency often associated with the use of antipsychotic medications. One study has reported an incidence rate of 0.2% for NMS among patients on neuroleptics [4]. It is characterized by a clinical syndrome of altered mental status, rigidity, fever, and autonomic dysfunction. First described in the 1960s, it is a rare psychiatric emergency that can be fatal if not recognized early and treated appropriately. An unadjusted mortality rate of 5.6% has also been reported [4]. Regardless of the underlying mechanism in NMS, central and peripheral manifestations eventually coalesce in a diffuse encephalopathic process that can masquerade as VE [5]. Encephalitis, an acute, inflammatory process affecting the brain, is most commonly caused by a viral infection [5]. Similar to NMS, the pathogenesis of HSV encephalitis (HSE) is poorly understood. HSE is considered the most important treatable viral encephalitis with a reported incidence rate of one case per million per year in the US [5]. Both direct virus-mediated cytotoxic effects and indirect immune-mediated processes have been implicated in a mechanism leading to neuronal death [6-7]. Brain infection is believed to result from the central neuronal transmission of the virus from a peripheral site to the brain via the trigeminal or olfactory nerve. If left untreated, HSE is equally often progressive and fatal in one to two weeks. In 1977, a study reported a 70% mortality in untreated patients [8]. As no pathognomonic clinical findings have been reported for HSE, a high index of suspicion is crucial to patient care, particularly in immunocompromised patients with febrile encephalopathy. Once HSE is suspected, the clinical evaluation must be expedited to confirm the diagnosis and commence treatment in order to reduce mortality.

In this article, the authors present the case of a medical patient presenting with neuropsychiatric symptoms, whose differential diagnoses included VE and an atypical form of NMS (Forme Fruste NMS). This case illustrates the significance of a comprehensive physical examination in the early diagnosis and appropriate treatment of a severely ill patient with a potentially fatal differential diagnosis.

## Case presentation

**Figure 1:**
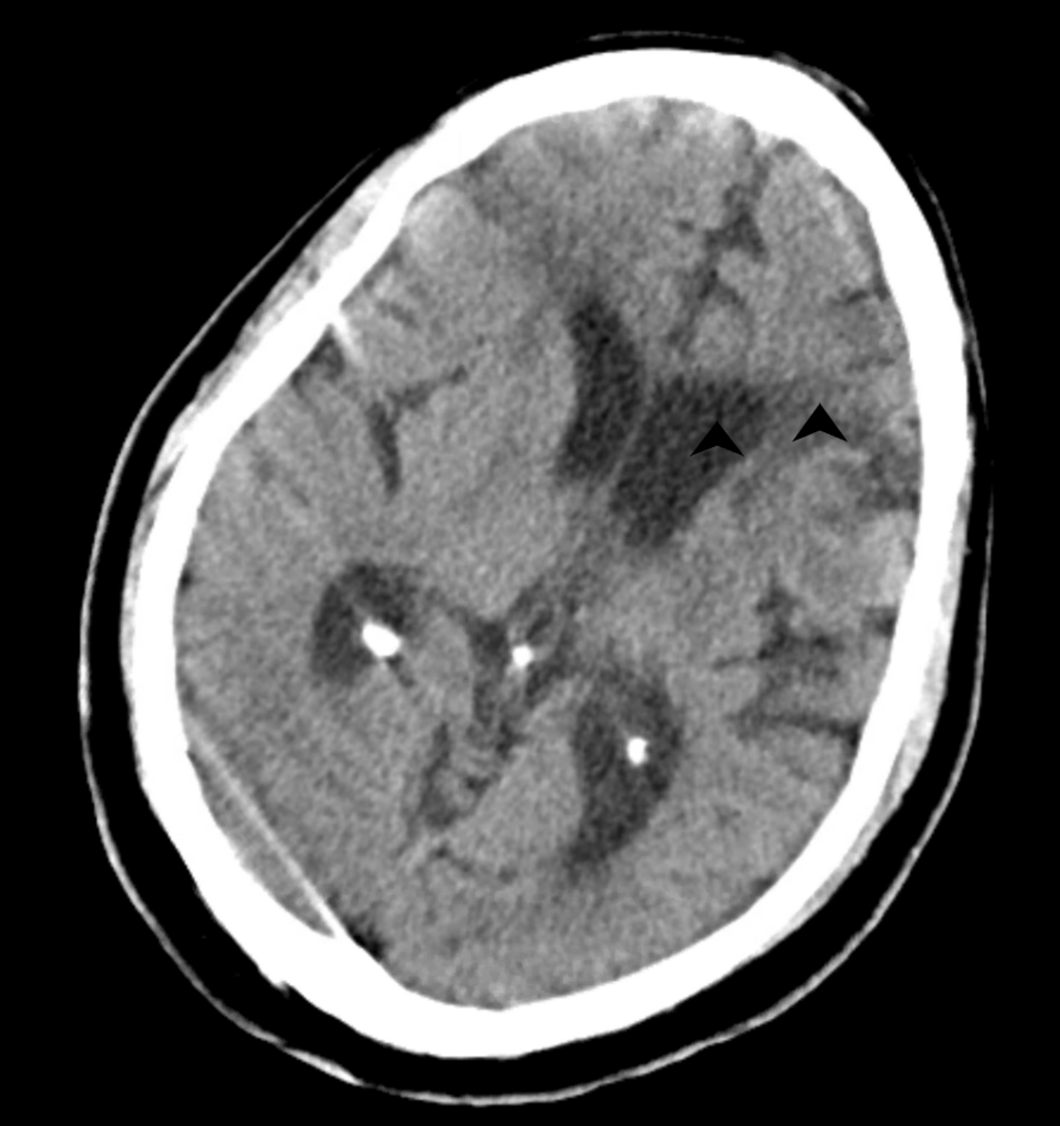
Non-contrast CT scan of the head showing enlarged left lateral ventricle and encephalomalacia in the left middle cerebral artery territory (black arrows).

A 59-year old male, with an unknown medical and psychiatric history, was brought to the emergency department (ED) by emergency medical service (EMS) after he was found wandering the streets and knocking randomly on people’s doors. The patient presented with an altered mental status and was oriented to name only. The history obtained from him was poor and unreliable, given his confused, irrational speech and dysarthria. He gave a pharmacy location as his home address and was unclear about his past medical history. There was no alternative historian available for collateral information. He was subsequently admitted to the medicine service where he was evaluated by the neurology and psychiatry services, respectively. His head CT scan revealed encephalomalacia in the left middle cerebral artery territory suggestive of previous cerebrovascular disease (Figure 1).

**Figure 2:**
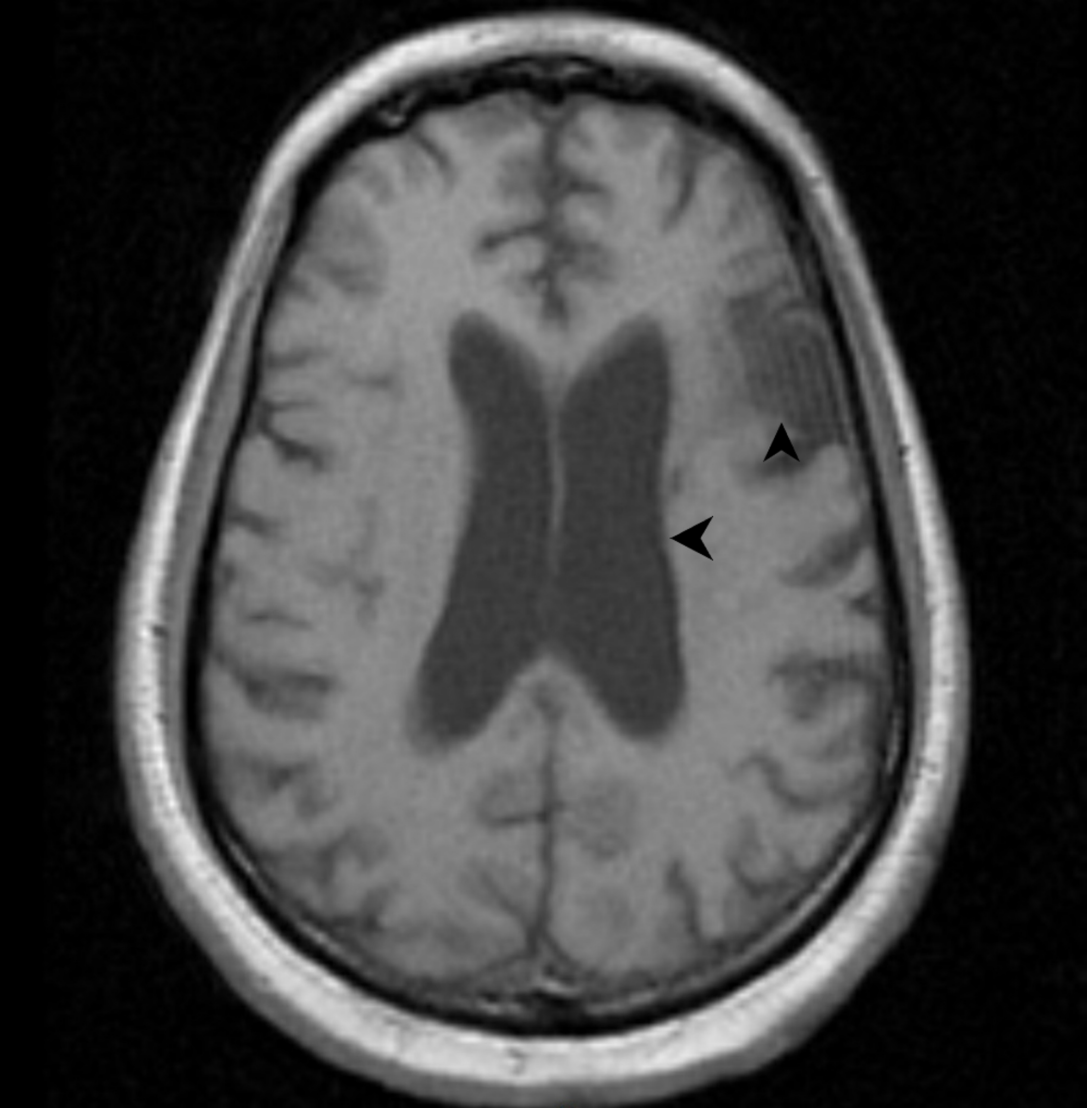
T1-weighted MRI scan of the brain (without contrast) also showing an enlarged left lateral ventricle and encephalomalacia in the left middle cerebral artery territory (black arrows).

Findings from the head CT scan were confirmed on the brain MRI done subsequently (Figure 2).

The patient’s erythrocyte sedimentation rate (ESR) and C-reactive protein (CRP) levels were both mildly elevated, at 81 mm/hour and 0.8 mg/dL, respectively. On initial psychiatric assessment, he was found to be a poor historian with dysarthria and confabulation as well as displaying poor insight into his illness. He was diagnosed with vascular dementia based on neurocognitive deficits and the neuroimaging finding of encephalomalacia. No acute psychiatric interventions were, however, recommended at the time.

The psychiatric consultation and liaison service were re-consulted three-and-a-half months later because the patient had an altercation with his roommate in the hospital, provoked by a bizarre delusion. He had believed that his roommate had exited the hospital through a bathroom door and had returned intoxicated, also through the same door. In reality, there was no avenue for egress or entry to the hospital through this route. This time, the psychiatric service recommended divalproex sodium 500 mg orally every 12 hours and risperidone 1 mg orally also every 12 hours. His delusions resolved, and there were no other acute psychiatric events for about two months.

**Figure 3:**
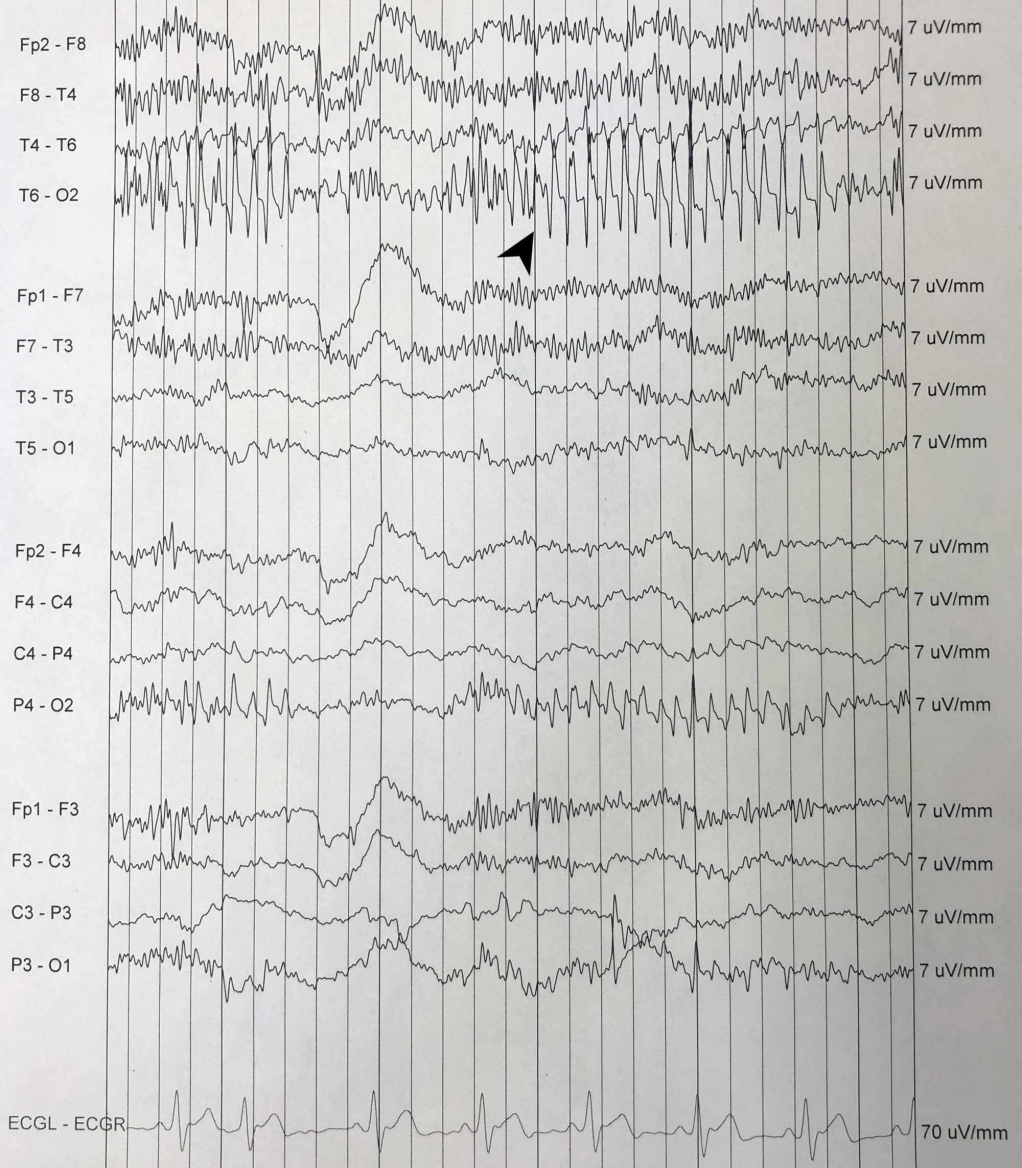
EEG from a routine portable encephalography (using 10-20 recording) showing superimposed spike activity (black arrow) over background slowing. EEG: electroencephalogram

He subsequently became agitated with another acute change in his mental status, and this time was noted to have muscle rigidity with a body temperature of 103°F. His white blood cell (WBC) count was elevated at 25,050/mm^3^, with elevated band neutrophils at 9%. His creatinine phosphokinase (CPK) was also elevated at 11, 627 U/L. Suspected to have NMS, his primary treatment team discontinued risperidone and again consulted psychiatry. At the follow-up psychiatric evaluation, he was noted to be shivering and had a diminished level of alertness. A physical examination done by the psychiatrist revealed some muscle rigidity that appeared to be voluntary. Electroencephalography (EEG) revealed mild background slowing with superimposed fast activity (Figure 3).

The lumbar puncture results included elevated cerebrospinal fluid (CSF) protein, CSF glucose, and CSF red blood cells (RBCs); CSF cultures were negative for all infectious agents. The patient, however, tested positive for immunoglobulin G (IgG) antibodies to HSV-1 and -2 on CSF analyses. His procalcitonin level of 4.81 ng/mL was also elevated. These results further pointed towards an infectious etiology, with HSV as the primary pathogen. For this reason, the low dose of risperidone, and the possibly voluntary muscle tone, the psychiatrist ranked atypical NMS low in his differential diagnosis.

After an initial temperature spike, the patient’s temperature subsequently ranged from 97.7-101.8 F before normalizing permanently. Over the course of his treatment with intravenous hydration and parenteral lorazepam, creatine phosphokinase (CPK) levels progressively decreased and eventually also returned to normal. He also completed an oral course of acyclovir to treat the possible HSV infection. No longer agitated and psychotic, he was deemed psychiatrically and medically stable to be discharged to a nursing home. Table 1 outlines the salient laboratory test values reported in this case.

**Table 1:** Reported laboratory test values for the presented case. CRP: C-reactive protein; ESR: erythrocyte sedimentation rate; WBC: white blood cell; CPK: creatine phosphokinase; CSF: cerebrospinal fluid; RBC: red blood cell

Test	Result	Normal Range
CRP	0.8 mg/dL	0.0-0.3 mg/dL
ESR	81 mm/Hour	0-30 mm/Hour
WBC	25,050/mm^3^	4.5-11.0/mm^3^
Band Neutrophils	9%	0-5.0%
CPK	11,627 U/L	38-244 U/L
Procalcitonin	4.81 ng/mL	<0.1 ng/mL
CSF Protein	64 mg/dL	15-45 mg/dL
CSF Glucose	81 mg/dL	40-70 mg/dL
CSF RBCs	208/mm^3^	0/mm^3^

## Discussion

HSE, the most common sporadic acute viral encephalitis in the western world [9], can present with sudden onset of fever, headache, altered mental status, autonomic dysfunction, and meningeal signs. Patients also present with focal neurological deficits such as hemiparesis, mutism, ataxia, pyramidal signs, involuntary movements, and seizures. Primary infection with HSV, resulting in HSE, occurs in only about one-third of cases while the rest occurs in patients with serologic evidence of a preexisting HSV infection, as in the index patient because his CSF analysis tested positive for IgG antibodies to HSV-1 and -2. The latter is typically due to the reactivation of latent peripheral infection in the olfactory or trigeminal neural system or reactivation of latent central infection in the brain [10].

The difficulty in maintaining a high suspicion for HSE lies in its nonspecific symptoms, as well as in its potential for the manifestation of psychiatric symptomatology. These may include agitation, personality changes, paranoia, hallucinations, and psychosis. Consequently, HSE is not always the first diagnosis that comes to mind to explain these manifestations, especially in a patient being treated by a psychiatrist. As evidenced by the index case, however, it is important that treating physicians cast a wide net to rule out other disorders with shared symptomatology [9]. Table 2 outlines some key facts about HSE [9].

**Table 2:** Herpes simplex encephalitis. EEG: electroencephalogram; HSE: herpes simplex encephalitis

The most common cause of non-endemic, acute fatal encephalitis in the Western world.
High level of clinical suspicion is necessary.
First central nervous system viral infection to be successfully treated with antiviral therapy.
One of the first to have routine cerebrospinal fluid polymerase-chain reaction diagnosis with high specificity and sensitivity.
Seen in neonates and adults.
Abrupt onset with frontotemporal features.
Initiation of treatment on clinical suspicion is required.
A combination of magnetic resonance imaging, EEG, and cerebrospinal fluid tests is usually diagnostic.
High mortality and morbidity in untreated patients.
Milder symptoms in atypical HSE.

NMS is an important differential diagnosis in cases with HSE. It is a life-threatening side effect of antipsychotic medication and is commonly associated with high-potency first-generation antipsychotics, such as haloperidol and fluphenazine. It has also been seen with the use of second-generation antipsychotics, such as risperidone and olanzapine, and even some antiemetic agents. Although symptoms are usually seen within the first two weeks of medication initiation or dose increase, it can still occur even after a single dose of the offending agent or after being on the same dose for years [11]. While VE describes an acute inflammation that diffusely affects the brain, NMS has been hypothesized to involve two main pathologic mechanisms - DA receptor blockade in the hypothalamus and interference with nigrostriatal dopamine pathways [12]. Multiple additional mechanisms have also been theorized to explain NMS but the pathogenesis of this condition remains poorly understood. It typically presents with a fever that can also be of sudden onset, altered mental status, muscle rigidity, and autonomic dysfunction [13]. Furthermore, like HSE, NMS symptoms can vary substantially from mild drowsiness and confusion to agitation, delirium, and coma [14]. Diagnostic criteria for NMS are presented in Table 3 [13].

**Table 3:** Neuroleptic malignant syndrome diagnostic criteria - expert panel consensus. * The mean priority score indexes each criterion according to its relative importance in making a diagnosis of neuroleptic malignant syndrome (NMS) according to an expert panel.

Diagnostic Criteria	Priority Score *
Exposure to dopamine antagonist, or dopamine agonist with withdrawal, within past 72 hours	20
Hyperthermia (> 100.4 F or > 38.0 C on at least 2 occasions, measured orally)	18
Rigidity	17
Mental status alteration (reduced or fluctuating level of consciousness)	13
Creatine kinase elevation (at least 4 times the upper limit of normal)	10
Sympathetic nervous system lability, defined as at least 2 of the following: Blood pressure elevation (systolic or diastolic > 25 percent above baseline) Blood pressure fluctuation (> 20mmHg diastolic change or > 25mmHg systolic change within 24 hours) Diaphoresis Urinary incontinence	10
Hypermetabolism, defined as heart rate increase (> 25 percent above baseline) AND respiratory-rate increase (> 50 percent above baseline)	5
Negative workup for infectious, toxic, metabolic, or neurologic causes	7
	Total: 100
No threshold score has been defined and validated for the investigators in making a diagnosis of NMS.	

Physicians treating patients with these symptoms should rule out central nervous system infections early, as delayed diagnosis can be life-threatening. Adopting a step-wise approach in the management of these patients would be clinically prudent. Initial non-invasive procedures, including an EEG and non-contrast brain imaging studies, are encouraged to rule out encephalitis. Where clinical suspicion for encephalitis remains high, obtaining a lumbar puncture becomes necessary to facilitate timely and appropriate treatment. Patients with HSE will have characteristic CSF findings: an elevated opening pressure, lymphocytosis, normal glucose levels, and raised protein levels. The most sensitive test for diagnosis is the CSF polymerase chain reaction for HSV. In comparison, patients with NMS will have normal CSF and EEG findings but will have an elevated CPK level, leukocytosis, elevated lactate dehydrogenase levels, electrolyte abnormalities, iron deficiency, and myoglobinuria [9].

Further complicating clinical differentiation between NMS and HSE is the existence of a rare “forme fruste” NMS [15]. Patients with this atypical presentation of NMS may display mild or no rigidity, may not have consistent hyperthermia or hypertension, and may have elevated CSF protein [15]. This type of NMS, with even fewer specific symptoms and test results than typical NMS, may result in some physicians gravely eliminating NMS from their list of differential diagnoses. It is important for treating physicians to widen the scope of possible etiologies, especially if a patient is on a low-dose, second-generation antipsychotic and has not had a dose change in months, as in the index case [16]. Table 4 summarizes some clinical conditions that can mimic NMS. This is why the consulting psychiatrist in the presented case maintained a clinical suspicion for NMS in spite of his atypical presentation, and why lorazepam was included in his treatment, in addition to acyclovir. Underscoring the importance of physical examinations in psychiatric patient encounters, the patient’s diagnosis was reconsidered only after the consulting psychiatrist performed one. The psychiatrist recommended broadening the differential diagnosis and encouraged further investigations based on the physical examination findings.

**Table 4:** Differential diagnosis for neuroleptic malignant syndrome. CSF: cerebrospinal fluid; CPK: creatine phosphokinase; MDMA: 3,4-methyl​enedioxy​methamphetamine, commonly known as ecstasy; EEG: electroencephalogram; EMG: electromyogram

Differential Diagnosis	Distinguishing Features
Infectious Meningitis or Encephalitis Brain Abscess Sepsis Rabies	History of prodromal viral illness, headaches or meningeal signs; Presence of seizures or localizing neurological signs; Brain imaging; CSF studies
Metabolic Acute Renal Failure Rhabdomyolysis Thyrotoxicosis Pheochromocytoma	Renal or thyroid function tests; Absence of neuroleptic treatment; Presence of severe hypertension; Significantly elevated catecholamines and metanephrines
Environmental Heat Stroke Spider Envenomations	History of exertion or exposure to high temperatures; Hot dry skin, skin lesion suggestive of spider bite; Absence of rigidity; Abrupt onset
Drug-Induced Malignant Hyperthermia Neuroleptic-induced Syndromes Parkinsonism Acute Dystonia Acute Akathisia Tardive Dyskinesia Postural Tremor Non-neuroleptic-induced Syndromes Serotonin Syndrome Anticholinergic Delirium Monamine Oxidase Inhibitor Toxicity Lithium Toxicity Salicylate Poisoning Strychnine Poisoning Drugs of Abuse (cocaine, amphetamine, methamphetamine, MDMA, phencyclidine)	History of use of inhalational anesthetics; Family history of malignant hyperthermia; Presence of hyperkinesias; Positive toxicology/drug-level screen; Low or normal CPK; Presence of nausea, vomiting, diarrhea; Presence of anticholinergic signs (dilated pupils, dry mouth, dry skin, urinary retention); Presence of rash, urticaria, or eosinophilia; History of drug dependence, abuse, or overdosages
Drug-withdrawal Syndrome Alcohol Benzodiazepines Baclofen Sedatives Hypnotics	History of drug dependence, abuse, or overdosages; Absence of neuroleptic treatment; Toxicology screen
Neurological or Psychiatric Disorder Parkinsonism Nonconvulsive Status Epilepticus Lethal Catatonia	Absence of fever or leukocytosis; Presence of hyperkinesias, later emergence of rigidity; Prior history of catatonic states; Absence of neuroleptic treatment EEG
Autoimmune Polymyositis	Proximal weakness; Abnormal EMG or muscle biopsy; Presence of cancer or interstitial lung disease

In a 2017 survey of the Royal College of Psychiatrists, researchers examined the frequency that psychiatrists performed physical examinations and reviewed potential modifying factors [17]. The results revealed that both postgraduate level of training and the setting of the patient encounter influenced whether or not a physical examination was conducted. Eighty-six percent of junior physicians reported they routinely performed physical examinations in an inpatient setting, which was considerably more than the 28% of senior physicians who did so. The study also revealed that both groups of physicians performed physical examinations far less frequently in the outpatient setting [17]. Refer to Table 5 for these statistics.

**Table 5:** Frequencies with which surveyed psychiatrists conducted physical examinations. PE: physical examination; Senior: consultant, non-consultant career grade, professor, lecturer or research fellow, or senior trainee; Junior: psychiatric core trainee or General Practitioner (GP) or foundation trainee * Data not reported

	N responded to an item	Senior	Junior	N (%)
Routinely perform PE on inpatients	2072	375/1324	294/340	682 (33.0)
How many PE on inpatients	685	*	*	*
1-2 per week		190	161	356 (58.6)
3-5 per week		89	92	185 (30.4)
6-10 per week		33	25	58 (9.5)
11-20 per week		4	4	8 (1.3)
More than 20 per week		1	0	1 (0.2)
Routinely perform PE on outpatients	2066	165/1530	35/338	203 (9.8%)
How many PE on outpatients	195	*	*	*
1-2 per week		83	14	98 (59.4)
3-5 per week		37	6	45 (27.3)
6-10 per week		15	3	18 (10.9)
11-20 per week		3	0	3 (1.8)
More than 20 per week		1	0	1 (0.6)

Some reasons for the dearth of physical examinations by psychiatrists include atrophy of skills, insufficient equipment, and concern about the way physical contact may psychologically impact patients [18]. While it is important to be aware of the latter, it has been argued that physical examinations by psychiatrists can actually strengthen the physician-patient relationship. When psychiatrists perform physical examinations, they establish themselves as physicians to the patients, build rapport, and reassure patients about the care they receive [18]. Psychiatrists, with physical examination findings, are better able to distinguish between physical disease, psychiatric illness, and medication side effects as the cause of a patient’s symptoms. Psychiatrists are also better able to establish a patient’s baseline and identify preexisting conditions that require tailored treatment plans [17]. More importantly, psychiatrists are uniquely positioned to advocate for psychiatric patients who often lack the social skills and resources to seek and obtain general medical care for themselves [18]. As the only physicians, many of these patients get to see, developing and maintaining physical examination skills as well as confidence in deploying them, are, therefore, crucial in a psychiatrist’s portfolio.

Stigma and unfamiliarity with mental illness may lead non-psychiatrist physicians to readily attribute changes in mental status and agitation in patients to a primary psychiatric diagnosis. Known as confirmation bias, this may be difficult to challenge in the absence of physical examination findings by psychiatrists. Confirmation bias is a type of cognitive bias that often influences a physician’s clinical decision making and judgment [19]. It is the tendency of an individual to focus on information that aligns with one’s preconceptions and to ignore information that defies them [19]. This can include a physician unintentionally misinterpreting or disregarding elements of laboratory results such that it fits with their presumed diagnosis. When physicians are influenced by this bias, it can lead to a narrow list of differential diagnosis that often excludes the correct diagnosis, causing treatment delay or preventing the appropriate treatment entirely. Confirmation bias can even be lethal, as in a case reported in the New England Journal of Medicine [20], where a patient with a past psychiatric history of anxiety was misdiagnosed as having a panic attack. Unfortunately, she rapidly deteriorated and went into asystole [20].

## Conclusions

Ultimately, it is important to recognize that cognitive biases are innate in all humans and are not limited to physicians. When a physician, however, fails to recognize confirmation bias, the impact can be deadly. Educating physicians to recognize and improve their awareness of this cognitive bias will improve diagnostic accuracy and, hopefully, translate into better patient care. Studies examining the attitude of psychiatrists in subspecialties towards physical examinations will also shed more light on this important aspect of patient care.

The authors recognize that confirmatory testing was not done for the presented case. The diagnosis of HSE was based on the detection of IgG antibodies to HSV-1 and -2 in the patient’s CSF, as opposed to confirmation via the polymerase chain reaction. Given the fact that he was successfully treated with lorazepam and acyclovir, it is also difficult to conclusively establish his diagnosis as NMS, HSE, or the co-occurrence of both.
